# Surgical Alternative for Treatment of Vertical Root fracture: A Case Report

**Published:** 2012-03-01

**Authors:** Emmanuel João Nogueira Leal da Silva, Gustavo Romão dos Santos, Renato Liess Krebs, Tauby de Souza Coutinho-Filho

**Affiliations:** 1. Piracicaba Dental School, State University of Campinas, Brazil; 2. UERJ, Brazil

**Keywords:** Diagnosis, Surgical Endodontic, Tooth Fracture, Treatment

## Abstract

Vertical root fracture (VRF) has been a great challenge in dentistry; most fractures often result in tooth extraction. Inflammation of tissues around the fractured root is the main reason for tooth extraction. Based on the strategic importance of some fractured teeth, treatment may be necessary and often complicated. However, performing a proper repair or even splinting the fractured segments may result in tooth preservation. Accordingly, in this case we report a new method for fractured tooth preservation. The surgical exposition of the fracture tooth was carried out through the radicular portion of the element via ultrasonic preparation, filling with composed resin and a synthetic hydroxyapatite graft. All these were performed around the tooth which received five sections of low-power laser. The patient was followed for two years with no signs or symptoms of inflammation and gingival recession. In conclusion, the used treatment protocol could be considered as a promising approach for VRF treatment, especially in cases where there is advanced or moderate bone loss in the surrounding sites of the fractured tooth.

## Introduction

Vertical root fracture (VRF) is an extremely compromising event for the tooth and heavily influences treatment protocol. Usually, this leads to loss of dental elements. The main factors that lead the treatment towards extraction of the fractured tooth are bacterial infiltration and subsequent inflammation in the fracture area, resorption of nearby alveolar process and being substituted with defensive cells [1].

Diagnosis of VRF is challenging and complicated; this is due to unclear signs and symptoms which are mostly without any clinical evidence [2]. Clinical characteristics of VRF can be presented with pain, edema, mobility, and periodontal diseases [3]. The prevalence of VRF has been reported to vary from 2 to 5% [2][4][5][6]. The etiology could be several iatrogenic factors e.g. post placement [7][8][9], excessive forces during the gutta-percha condensation [10][11], excessive masticatory forces [12], and extensive dental preparations [2][13]. Although in some cases of VRF, the causes are unclear [3].

Some therapeutic alternatives have been studied for the VRF looking in order to introduce more conservative treatment for these cases. Mineral trioxide aggregate (MTA) [14], bone grafts [15], hydroxyapatite grafts [16], laser therapy [17], glass-ionomer cement [18], dentin-bonded resin [19][20], and other materials have been already used for stabilizing the bone loss associated with VRF.

The aim of this case report was to present a successful treatment of a VRF using dentin-bonded resin to fill outer space of the fracture.

## Case report

A 58-year-old female with no contributing medical history was referred to the Endodontic service of the Universidade do Estado do Rio de Janeiro, complaining about pain in the region of the upper right canine tooth.

In the clinical examination, edema was verified in the buccal and apical region and no periodontal pocket was observed. Radiographic examination revealed a previous endodontic treatment. The first treatment option consisted non-surgical retreatment following by inspection with surgical microscope.

**Figure 1:**
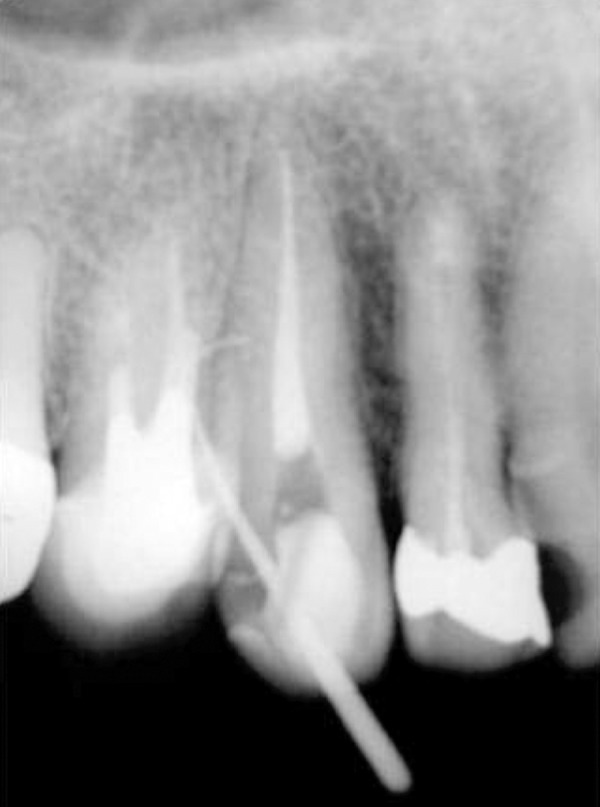
Tracking test in the gingival fistula

Root canal filling materials were removed and after the root canal preparation, a meticulous inspection with the surgical microscope was accomplished (D.F. Vasconcellos, Rio de Janeiro, Brazil). No alteration in the internal walls of the tooth was observed. The root canal was then filled and the patient was followed-up after 7 days. She complained of persistent local sensibility. Clinically, edema and pus were observed draining in the vestibular of inserted gum. Also, a 10 mm deep periodontal pocket was observed, and after a tracking test in the gingival fistula (Figure 1), we opted for an exploring surgery to detect the origin of the pus.

**Figure 2:**
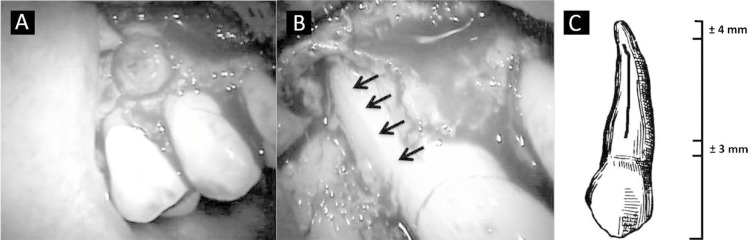
A) Granulation Tissue; B) VRF in the vestibular face of the tooth; C) Schematic fracture drawn

A full-thickness flap was elevated and a granulation tissue was observed at the root buccal surface; also, a total absence of the alveolar bone was observed in this area all through the tooth length except for 3 mm of the radicular apex (Figure 2A). After removing the inflammatory tissues, it was possible to verify the presence of a VRF in the buccal surface of the tooth (Figure 2B). A schematic drawn shows the fracture in detail (Figure 2C).

**Figure 3:**
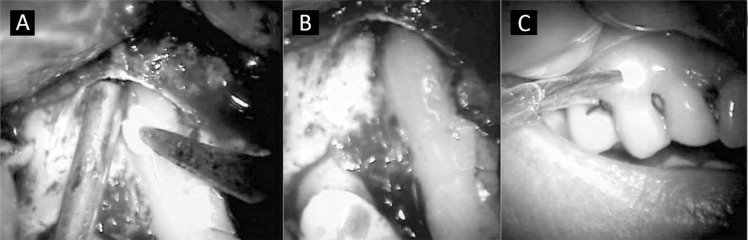
A) Filling of the cavity with composite resin; B) Cavity with composite resin; C) Low Power laser treatment

Since the clinical crown of the tooth was in excellent condition, the patient was notified about tooth extraction as the common treatment in these cases, but she could opt for a more conservative treatment to maintain the tooth, but with no guarantee of success. It was then decided to explore the full extent of the fracture, by complete exposing using an ultrasonic unit (Mini Endo Excellence in Endodontics, USA) and ultrasonic tips for surgery. Then it was made a further 1 mm throughout the length of incomplete fracture. Acid etch was performed for 30 seconds. The acid was removed by vacuum suction followed by another washing with 0.9% saline solution and drying with sterile gauze. Single Bond adhesive (3M, Sao Paulo, Brazil) was then used and it was light cured for 20 seconds. After adhesive polymerization, we filled the cavity with composite resin Tetric Ceran (Ivoclar, Schaan, Liechtenstein), followed by light curing for 40 seconds (Figure 3A and Figure 3B).

Due to the large bone fenestration and possible gingival recession, was decided to fill the bone fenestration with a synthetic hydroxyapatite graft (Intra-Lock, Boca Raton, FL, USA). After accommodation of the graft, the flap was repositioned and sutured with 4.0 silk. In order to accelerate tissue repair, minimize possible gingival recession and an anti-inflammatory response after the surgery, 5 sections of low power laser with a wavelength of 685 nm (Photon Lase, DMC, Sa˜o Carlos, Brazil) was performed around the tooth with occasional applications at mesial, distal, apical and cervical with intervals of 48 hours between sessions (Figure 3C).

**Figure 4:**
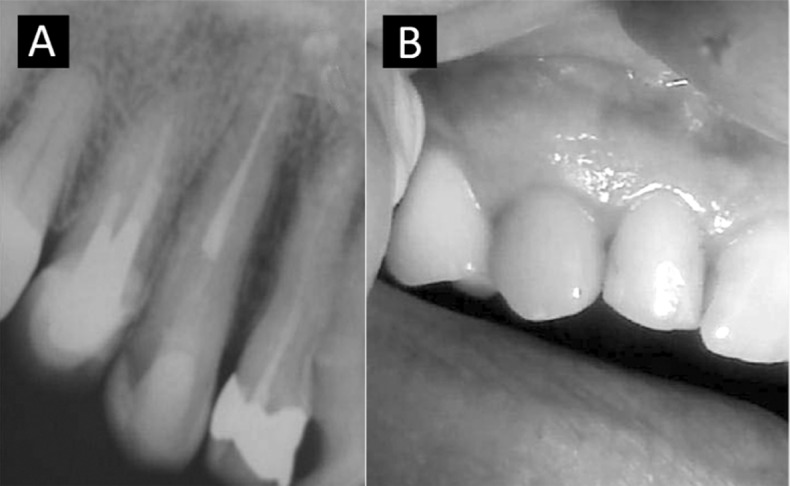
Two years follow-up; A) Radiographic; B) Clinical

The patient was followed for two years (Figure 4A and Figure 4B). Clinical and radiographic healthy status was observed with no signs or symptoms of inflammation and gingival recession.

## Discussion

Tooth fracture has been described as a major complication in dentistry and is the third most common cause of tooth loss, after dental caries and periodontal disease [21]. A VRF extends to the periodontal ligament, and soft tissue grows into the fracture. Separations between fractured fragments increase and resorption areas enlarge over time, negatively affecting the possibility of further treatment in the affected area [22]. Rapid diagnosis of a VRF is required and important to prevent additional bone loss that will further impede reconstruction.

The final diagnosis of VRF is, at times, difficult, but quick decision making is necessary to stop rapid bone loss once the fracture communicates with the oral environment [23]. A definitive diagnosis of VRF can be achieved by an exploratory flap, as done in the present study. The treatment of complete or incomplete vertical fracture is healing the disease process and preventing the progress of the resorption of the alveolar bone. The procedure indicated in these cases is the extraction of the fractured tooth contaminated and thus eliminating the pathological agent. Considering that it is a radical step, because of its irreversibility, some conservative alternatives have been studied in order to preserve the fractured element, maintaining the function and the healthy appearance [14][18][24][25][26], as done in this study.

MTA has been used as a biomaterial for reconstruction of the fractured area [14]. The properties of the MTA and its biocompatibility with periradicular tissues, low toxicity, good marginal sealing, hydrophilicity and high pH [27][28][29], make it an effective material in treatment of root fractures; however, poor handling and high setting time of this material make it hard to be used in some cases.

Attempts for conservative treatment of teeth with VRF also reported as treatment of 26 vertically fractured roots using replantation and reconstruction with 4-methacryloxyethyl trimellitic anhydride in methyl methacrylate initiated by tri-n-butyl borane dentin bonded resin [30]. The authors found that 18 cases were functional and retained and 6 were fully successful after observation periods ranging from 4 to 76 months. They found that teeth with longitudinal fractures extending more than two-thirds from the cervical portion toward the apex and posterior teeth showed significantly lower longevity. In the present study the tooth showed incomplete VRF without separation of segments.

In this case we used a composite resin which showed success after a period of two years for follow-up. The choice of resin was based on several previous studies that showed the effectiveness of resin materials in the reconstruction of teeth with VRF [19][20][22][23][24][25][26]. In several studies the periodontal condition was monitored clinically and radiographically for a long time, and the results were promising.

Oztürk and Unal, reported a case of complete VRF in the tooth that was extracted, the fracture was restored with resin cement, and then implanted in the alveolus [31]. In the present report we chose to not extract the tooth, relying on the results of the previous study [19] that noted no statistical difference when the reconstruction of teeth with FRV was performed after extraction tooth or with the unit held in its socket.

Hayashi et al., reported no failure in vertically fractured incisors treated with this method, although failures did occur in premolars and molars [20]. The authors suggested that the posterior teeth were negatively affected by strong occlusal forces. The morphology and location of anterior teeth also facilitate the maintenance of gingival health, a further reason for positive outcomes after VRF treatment. The authors also reported that success in treatment of VRF is defined as clinically acceptable when the tooth showed no clinical symptoms, demonstrating periodontal tissue healing/regeneration, and improvements in periodontal probing at the fracture site. According to these criteria, this case report presents success after a period of two years of follow-up.

The overall esthetic result has recently become a fundamental factor in the evaluation of treatment success [32][33]. In the present case, it was considered of utmost importance, because the teeth were located in the anterior maxilla. At 24 months follow-up, the tooth was functional and the patient was satisfied with the esthetics.

Further research and better knowledge about alternative treatments for complete and incomplete root fractures are needed to provide the basis for a more accurate and reliable evidence, in order to establish a more conservative solutions and thus avoiding the loss of the fractured element.

## Conclusions

In conclusion, the treatment protocol used in this case is a promising approach for VRF treatment, especially in cases where there is advanced or moderate bone loss in the surrounding sites of the fractured tooth.
